# Astrocyte Reactivity Following Blast Exposure Involves Aberrant Histone Acetylation

**DOI:** 10.3389/fnmol.2016.00064

**Published:** 2016-08-08

**Authors:** Zachary S. Bailey, Michael B. Grinter, Pamela J. VandeVord

**Affiliations:** ^1^Department of Biomedical Engineering and Mechanics, Virginia TechBlacksburg, VA, USA; ^2^Salem Veterans Affairs Medical CenterSalem, VA, USA

**Keywords:** blast neurotrauma, neuroinflammation, histone acetylation, brain injury, epigenetics

## Abstract

Blast induced neurotrauma (BINT) is a prevalent injury within military and civilian populations. The injury is characterized by persistent inflammation at the cellular level which manifests as a multitude of cognitive and functional impairments. Epigenetic regulation of transcription offers an important control mechanism for gene expression and cellular function which may underlie chronic inflammation and result in neurodegeneration. We hypothesize that altered histone acetylation patterns may be involved in blast induced inflammation and the chronic activation of glial cells. This study aimed to elucidate changes to histone acetylation occurring following injury and the roles these changes may have within the pathology. Sprague Dawley rats were subjected to either a 10 or 17 psi blast overpressure within an Advanced Blast Simulator (ABS). Sham animals underwent the same procedures without blast exposure. Memory impairments were measured using the Novel Object Recognition (NOR) test at 2 and 7 days post-injury. Tissues were collected at 7 days for Western blot and immunohistochemistry (IHC) analysis. Sham animals showed intact memory at each time point. The novel object discrimination decreased significantly between two and 7 days for each injury group (*p* < 0.05). This is indicative of the onset of memory impairment. Western blot analysis showed glial fibrillary acidic protein (GFAP), a known marker of activated astrocytes, was elevated in the prefrontal cortex (PFC) following blast exposure for both injury groups. Analysis of histone protein extract showed no changes in the level of any total histone proteins within the PFC. However, acetylation levels of histone H2b, H3, and H4 were decreased in both groups (*p* < 0.05). Co-localization immunofluorescence was used to further investigate any potential correlation between decreased histone acetylation and astrocyte activation. These experiments showed a similar decrease in H3 acetylation in astrocytes exposed to a 17 psi blast but not a 10 psi blast. Further investigation of gene expression by polymerase chain reaction (PCR) array, showed dysregulation of several cytokine and cytokine receptors that are involved in neuroinflammatory processes. We have shown aberrant histone acetylation patterns involved in blast induced astrogliosis and cognitive impairments. Further understanding of their role in the injury progression may lead to novel therapeutic targets.

## Introduction

Mild traumatic brain injury (TBI) has been deemed the “signature” wound of recent military conflicts (Hoge et al., 2008). During Operation Iraqi Freedom and Operation Enduring Freedom, 78% of injuries were attributable to an explosive mechanism (Owens et al., 2008) leading to increased incidences of blast induced neurotrauma (BINT). BINT is a debilitating injury that absorbs a substantial amount of annual healthcare costs (Bilmes and Stiglitz, 2006). The injury is characterized by diffuse neurological damage that leads to long-term cognitive impairments.

The molecular sequelae of the injury involves accumulation of reactive oxygen species that overwhelm and deplete endogenous antioxidant systems. The compromise of antioxidant systems leaves the brain in a pro-oxidative environment causing mitochondrial damage, altered metabolism, and cell membrane damage (Cernak et al., 2001; Wang et al., 2011; Ahmed et al., 2013; Cho et al., 2013; Tümer et al., 2013; Shetty et al., 2014). Perivascular accumulation of reactive oxygen species can be detrimental to blood brain barrier (BBB) integrity through degradation of tight junctions (Shetty et al., 2014). A leaky BBB has been reported following BINT which can cause cerebral edema, initiation of apoptotic cascades in the surrounding tissue and the onset of neuroinflammation (Shapira et al., 1993; Readnower et al., 2010; Rubovitch et al., 2011; Easton, 2012; Abdul-Muneer et al., 2013). Pre-clinical studies have focused on elucidating the temporal response of the injury and observed sustained glial cell activation, apoptosis, and cognitive impairments. Histological and neurochemical analyses have revealed sustained neurological damage involving continued neurodegeneration and inflammation 3 months following injury (Sajja et al., 2015).

The inflammatory response following BINT has been characterized by diffuse astrogliosis (Cernak et al., 2011; Kamnaksh et al., 2011; Svetlov et al., 2012; Turner et al., 2013; Sajja et al., 2015, 2016). Under normal circumstances, astrocytes play important roles in modulating the BBB, neuronal circuit and synapse reorganization, and the inflammatory response (Burda et al., 2015; Sajja et al., 2016). In acute stages of injury astrocyte activation serves neuroprotective efforts, but chronic activation has been linked to neurodegeneration and memory deficits (Wyss-Coray and Mucke, 2002; Johnson et al., 2013; Sajja et al., 2015). Brain regions, including the hippocampus, prefrontal cortex (PFC), and amygdala, exhibit persistent astrocyte activation throughout the injury progression. Since these regions serve critical roles in proper memory function and anxiety, the continued inflammation likely contributes to the pathological anxiety and memory loss.

Astrocytes’ role in central nervous system is facilitated by coordinating cell to cell interactions through production of various signaling molecules. These molecules are critical for eliciting either pro- or anti-inflammatory response in the surrounding tissue environment but are also able to regulate BBB permeability (Sofroniew and Vinters, 2010; Sajja et al., 2016). Through secretion of various vasoactive molecules, astrocytes modulate BBB permeability which can lead to edema and inflammation (Sofroniew and Vinters, 2010). Inflammation is further regulated through specific cytokine production and secretion. Studies have shown cytokine dysregulation throughout the brain while also depicting injury induced inflammation (Kamnaksh et al., 2011; Dalle Lucca et al., 2012; Cho et al., 2013). These studies have demonstrated the involvement of molecules including various interleukins, tumor necrosis factor-alpha (TNF-α), and interferon-gamma (IFN-γ; Kamnaksh et al., 2011; Dalle Lucca et al., 2012; Sajja et al., 2012; Cho et al., 2013; Sajja et al., 2014b).

It has been reported, that both astrocyte activation and cytokine production involve regulation by histone acetylation (Thompson and Van Eldik, 2009; Correa et al., 2011; Kanski et al., 2014). Histone acetylation is an epigenetic process that regulates gene transcription through chromatin organization. Acetylation of histone proteins (H2a, H2b, H3, and H4) occurs on lysine residues within the N-terminal tail (Morales and Richard-Foy, 2000). Addition of the acetyl group neutralizes the positive charge and decreases the histone affinity to DNA (Grant, 2001). This creates a relaxed chromatin state in which the DNA is more accessible to transcription factors and transcription is promoted. Levels of acetylation of histone proteins are maintained through dynamic equilibrium between histone deacetylase enzymes (HDACs) and histone acetyltransferase enzymes (HATs; Eberharter and Becker, 2002). HATs facilitate the addition of acetyl groups and gene expression while HDACs facilitate their removal and subsequent gene repression. Histone acetylation has been implicated in both neuroinflammatory processes (Correa et al., 2011; Kanski et al., 2014), and in memory formation (Levenson et al., 2004; Vecsey et al., 2007; Valor et al., 2011). Thus, the hypothesis of this study is that BINT leads to aberrant histone acetylation patterns that underlie astrocyte activation and cognitive impairments.

To date, no studies have investigated histone acetylation abnormalities within the BINT pathology. However, few studies have investigated histone acetylation alterations using *in vivo* models of impact TBI. Controlled cortical impact and fluid-percussion models of TBI have been shown to induce acute histone hypo-acetylation in the hippocampus (Gao et al., 2006; Zhang et al., 2008). Studies have also focused on evaluating the effects of histone deacetylase inhibitor administration. These studies have demonstrated that histone deacetylase inhibitors decrease inflammation and improve cognitive and functional outcomes following impact TBI (Zhang et al., 2008; Dash et al., 2010). Taken together, these results suggest a role for histone acetylation within the injured brain that may appear to affect both neuroinflammation and the manifestation of cognitive impairments.

While studies have demonstrated the importance of histone acetylation following impact-induced TBI, changes in the histone acetylation need to be explored to help elucidate the mechanism of BINT. The work here provides an in depth investigation of histone changes resulting from blast exposure. For the first time, we provide evidence of epigenetic changes associated with blast induced astrogliosis. Since injury severity has been shown to elicit different inflammatory responses, we investigated whether severity-dependent histone alterations correlated with different cellular responses. In addition, we investigated whether neuropathology within the PFC correlated with memory impairments. A better understanding of the molecular changes following BINT will aide in determining the prognosis and identifying novel therapeutic intervention strategies.

## Materials and Methods

### Animals and Blast Exposure

**Figure 1:**
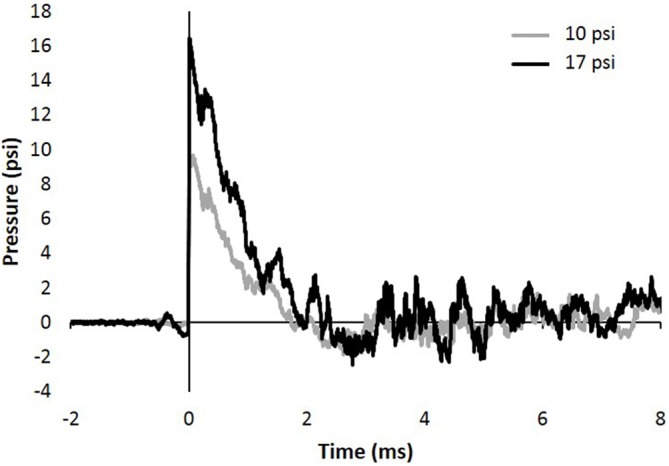
**Representative 10 and 17 psi blast curve profiles from Advanced Blast Simulator (ABS).** The curve is characterized by near instantaneous rise in pressure followed by an exponential decay and a brief negative phase before returning to ambient pressure. Animals were exposed to a single peak overpressure representing a free-field blast exposure.

The study described herein was carried out in accordance with experimental protocols approved by the University Institutional Animal Care and Use Committee at Virginia Tech. Prior to any experimentation, male Sprague Dawley rats weighing approximately 250–300 g (Envigo, Dublin, VA, USA) were acclimated for several days (12 h light/dark cycle) with food and water provided *ad libitum*. The blast wave was generated using a custom Advanced Blast Simulator (ABS; 200 cm × 30.48 cm × 30.48 cm). The ABS consists of three distinct sections to create, develop, and dissipate the blast wave. The blast wave develops following helium-driven rupture of calibrated acetate membranes. A passive end-wave eliminator was located downstream of the test location to facilitate the dissipation of the blast wave through a series of baffles. As a result, the test location is exposed to single peak overpressure representing a free-field blast exposure. Pressure measurements were collected at 250 kHz using a Dash 8HF data acquisition system (Astro-Med, Inc, West Warwick, RI, USA). Representative pressure profiles are shown in Figure 1. Analysis of pressure profiles was conducted using a custom Matlab script to calculate impulse and duration of the positive and negative phases and rise time. Peak overpressure was determined using the Rankine—Hugoniot relations and observed wave speed at the animal test location in the ABS.

Animals were randomly divided into three groups (*N* = 8/group): sham, 10 psi (70 kPa), 17 psi (119 kPa). Prior to blast exposure, animals were anesthetized with 3% isoflurane and placed in the ABS. Each animal was supported in the prone position inside the ABS facing the oncoming shock front using a mesh sling. The sling was designed to minimize flow hindrance and isolate primary blast injury by eliminating acceleration/deceleration injuries. This injury model has been previously shown to include minimal lung injury at the pressure levels being tested (Sajja et al., 2015). In order to assess changes specific to injury severities, tests were conducted at two blast pressures. Blast parameters are listed in Table 1. Changes in the magnitude of the blast wave have been previously shown to trigger different responses at the cellular and molecular level (Sajja et al., 2014a). Sham animals received the same procedures with the exception of blast wave exposure. Following sham or blast procedure, animals were observed through the recovery stages of injury and anesthesia. Animals were anesthetized with 3% isoflurane and perfused transcardially with phosphate buffered saline (PBS) 7 days following sham or blast procedures. Brains were extracted and sectioned in half along the midline. Half of the brain was prepared for immunohistochemistry analysis (IHC), the other half underwent microdissection to isolate PFC tissue. For IHC analysis, brains were immediately stored in 4% paraformaldehyde. Tissue for protein extraction was placed on a cold brain matrix and cut into 2 mm coronal sections. Sections were immediately frozen on solid CO_2_ and brain punches were taken from the medial PFC (mPFC).

**Table 1:** **Analysis of blast curve for each injury group**.

	10 psi	17 psi
Peak pressure (psi)	9.93 ± 0.90	16.33 ± 2.11
Positive duration (ms)	1.73 ± 0.05	2.25 ± 0.06
Positive impulse (psi * ms)	6.96 ± 0.76	12.21 ± 1.76
Rise time (ms)	0.04 ± 0.03	0.03 ± 0.02
Negative duration (ms)	1.58 ± 0.29	0.92 ± 0.07
Negative impulse (psi * ms)	1.11 ± 0.28	0.94 ± 0.08

*The profiles were broken into two phase: positive and negative. Impulse was defined as the integral of each phase, duration was the length of time for each phase. Data expressed as mean ± SD*.

### Novel Object Recognition Test (NOR)

Blast induced short-term memory impairments were evaluated using the Novel Object Recognition (NOR) test. Animals were acclimated to the NOR arena (80 cm × 80 cm) and room prior to blast or sham procedure. The NOR test was administered 2 and 7 days following blast exposure. The test consisted of two phase: T1 (familiarization phase) and T2 (testing phase). During T1, the animal was placed into the arena and allowed to familiarize with two identical objects for 5 min. For T2 the animal was placed into the same arena with one of the objects replaced with a novel object and allowed to explore for another 5 min. Novel object location was randomly assigned between the two object locations. The T2 phase occurred 20 min following T1 phase to elicit short-term memory response. In order to preserve novelty between time points, different novel objects were used during the 2 day and 7 day tests. For all tests, the experimenter left the room immediately after the start of each trial to avoid potential influences on behavior. The arena was cleaned following each trial.

All trials were recorded and tracked at 30 frames per second using EthoVision XT (Noldus Information Technology, Leesburg, VA, USA). Three point tracking was performed and included tracking of the nose-point, center of body, and base of the tail. The animals’ preference to explore the novel object was assessed using a discrimination index which represents the fraction of time spent at the novel object (Equation 1). Exploration was measured when the nose-point entered a 4 cm zone surrounding each object. The total time spent within these zones was used to calculate the discrimination index. Accurate tracking of each trial was confirmed through video analysis by an investigator blind to trial and treatment information.

(1) $$\text{Discrimination Index}=\frac{{\text{t}}_{\text{novel}\;\text{object}}}{{\text{t}}_{\text{novel object}}+{\text{t}}_{\text{familiar object}}}$$

Performance on the test was compared to a discrimination index of 0.5 which represents equal time spent at both objects. Due to the animals’ innate exploratory behavior, intact memory was interpreted as increased time spent at the novel object (Discrimination Index > 0.5). Poor discrimination of the novel object has been previously shown to reflect damage to memory formation (Reger et al., 2009; Ennaceur, 2010). Therefore, memory deficit was interpreted by the animals’ inability to discern the novel object (Discrimination Index ≤ 0.5).

Animals were excluded from further analysis if the animal demonstrated greater than 90% side preference during the T1 phase as a spatial preference to the arena will likely mask any memory driven behavior. Side preference was determined through comparison of time spent at the two identical objects. A 10 s minimum combined exploration time was required for inclusion in the analysis to avoid animals with reduced activity levels.

### Protein Extraction and Western Blot Analysis

Histone and cytoplasmic proteins were extracted as described by Rumbaugh and Miller (2011). Briefly, PFC tissue was homogenized in a Dounce homogenizer. The homogenate was centrifuged and the supernatant was saved as the cytoplasmic protein fraction. The pellet was resuspended in sulfuric acid to isolate histone proteins. Histones, being basic proteins, are acid soluble while most proteins precipitate out of sulfuric acid solution. Acetone was used to precipitate the histones, which were then resuspended in a buffer containing 10 mM Tris-HCl and protease inhibitor cocktail (Sigma Aldrich, St. Louis, MO, USA). This protocol has been proven effective for isolating histone proteins while preserving their posttranslational modifications (Rumbaugh and Miller, 2011). Protein concentrations were determined by Bradford Colorimetric assay (BCA; Thermo Fisher Scientific, Waltham, MA, USA), following the manufacturers protocol. Samples were stored at −80°C until Western blot analysis.

A capillary-based Western blot analysis (Simple Western) was performed using the WES system (ProteinSimple, Santa Clara, CA, USA). Protein samples were diluted and prepared by manufacturers’ protocol. The samples were denatured at 95°C for 10 min. After loading the plate, electrophoresis separation, antibody incubation, and chemiluminescence detection was carried out within the WES system using default settings. Specific antibodies for glial fibrillary acidic protein (GFAP; Abcam, Cambridge, UK), Ionized Calcium-Binding Adapater Molecule 1 (IBA-1; Biocare Medical, Concord, CA, USA), H2a (Cell Signaling Technologies, Danvers, MA, USA), H2b (Cell Signaling Technologies, Danvers, MA, USA), H3 (Cell Signaling Technologies, Danvers, MA, USA), H4 (Cell Signaling Technologies, Danvers, MA, USA), acetyl-H2a (AH2a; Cell Signaling Technologies, Danvers, MA, USA), acetyl-H2b (AH2b; Cell Signaling Technologies, Danvers, MA, USA), acetyl-H3 (AH3; Cell Signaling Technologies, Danvers, MA, USA), and acetyl-H4 (AH4; Cell Signaling Technologies, Danvers, MA, USA) were used.

Area under each electropherogram peak of interest was calculated using Compass software (ProteinSimple, Santa Clara, CA, USA). Peak fits were confirmed through analysis by an investigator blind to treatment. Expression for GFAP and IBA-1 was normalized to actin. Since actin was not present in the histone extracts, all histone expression was normalized to total H4 protein (Rumbaugh and Miller, 2011).

### HDAC Activity Assay

In order to investigate HDAC function, we evaluated nuclear HDAC activity levels. Nuclear protein extracts were obtained using a commercial kit, following the manufacturers’ protocol (Epigentek, Farmingdale, NY, USA). Samples were homogenized in lysis buffer using a Dounce homogenizer. Following centrifugation, the supernatant was removed and saved as the cytoplasmic protein fraction. The pellet containing cell nuclei was resuspended and sonicated. Cellular debris was spun down and nuclear proteins were isolated. Proteins were quantified using a BCA (Thermo Fisher Scientific, Waltham, MA, USA).

HDAC activity was measured using a commercial activity assay, following the manufacturers’ protocol (Millipore, Billerica, MA, USA). Briefly, samples were incubated in substrate solution allowing for deacetylation of the colorimetric substrate. Samples were washed and incubated with activator solution to cleave the colorimetric molecule from the deacetylated substrate. The absorbance was measured at 405 nm and compared between groups.

### Immunohistochemistry (IHC) Analysis

Brains were fixed in 4% paraformaldehyde and dehydrated in 30% sucrose solution prior to sectioning. Fixed brains were embedded and frozen in optimal cutting temperature medium (Sakura Finetek Inc., Torrance, CA, USA). Coronal sections (40 μm) were prepared in a cryostat microtome (Thermo Scientific Inc., Waltham, MA, USA) and stored in PBS at 4°C prior to staining procedures.

IHC was performed on randomly selected PFC cortex sections (*N* = 3 sections/animal, 8 animals/group) containing the anterior cingulate cortex (ACC) to investigate GFAP and AH3 co-localization. Tissue sections were rinsed with PBS and incubated in 3% bovine serum albumin blocking buffer for 1 h. Sections were then incubated in primary antibody diluted in blocking buffer overnight at 4°C. Specific antibodies for GFAP (Invitrogen, Carlsbad, CA, USA) and AH3 (Cell Signaling Technology, Danvers, MA, USA) were used. Samples were washed with PBS prior to incubation with secondary antibodies Alex Fluor 555 anti-rabbit IgG and fluorescence-tagged fluorescein isothiocyanate anti-rat IgG. Both secondary antibodies were used at 1:500 dilution in blocking buffer. After being washed with PBS, sections were mounted on slides, air-dried, and coverslipped with prolong antifade gold reagent with 6-diamidino-2-pheylindole (DAPI; Invitrogen, Carlsbad, CA, USA). Sections were examined using a Zeiss fluorescence microscope and images were captured with an AxioCam ICc1 camera (Zeiss, Jena, Germany). Three regions of interest within the ACC were imaged at 20× magnification for each section. Within each area, images were taken using three different fluorescent filters to capture GFAP, AH3, and DAPI signal. An average intensity value was derived from a total of nine images (three regions of interest, three coronal sections) for each animal. To confirm co-localization, randomly chosen slides were imaged using a Confocal microscope at 63× magnification (Zeiss, Jena, Germany).

Images were processed and quantified using ImageJ software (National Institute of Health, Bethesda, MD, USA). Corresponding GFAP and AH3 images were merged. Background was subtracted using the built-in ImageJ function. Composite images were then thresholded to isolate only pixels with both GFAP and AH3 expression. Green (GFAP) and red (AH3) fluorescent intensity of the resultant image was measured. AH3 signal was used to assess AH3 levels of astrocytes.

### RNA Extraction and Reverse-Transcription Polymerase Chain Reaction (RT-PCR) Analysis

Total RNA was extracted from PFC tissue using Trizol (Invitrogen, Carlsbad, CA, USA). RNA samples were incubated with DNAse to facilitate digestion of any residual DNA contamination (Promega, Durham, NC, USA). RNA samples were further purified using RNeasy MiniKit, following the manufacturers’ protocol. RNA purity and concentration was determined through UV measurements at 230 nm, 260 nm, and 280 nm. RNA purity was considered suitable when the 260/280 ratio was 1.8–2.0 and the 260/230 ratio was above 1.7.

To create a complimentary DNA template, RNA was incubated at 65°C with random hexamers and equimolar deoxynucleotide solution containing dATP, dCTP, dGTP, and dTTP. First-strand buffer, DTT solution, and RNAse inhibitor was added to solution and allowed equilibrate to 37°C. Reverse transcriptase was added to facilitate conversion of RNA to cDNA template. Based on manufacturers’ recommendation, 0.5 μg of RNA was used for conversion.

Polymerase chain reaction (PCR) arrays were used to evaluate expression patterns of inflammatory cytokines and cytokine receptors (Qiagen, Hilden, Germany). PCR arrays were conducted according to manufacturer’s protocol and were run on 7300 Real-Time PCR System (Applied Biosystems, Foster City, CA, USA) using the following protocol: 1 cycle (95°C for 10 min), 40 cycles (95 °C for 15 s, 60 °C for 1 min). PCR arrays were run in duplicate for two sham animals and two animals within the 17 psi injury group. Expression levels were normalized to hypoxanthine phosphoribosyltransferase 1 (HPRT1).

### Statistics

#### NOR Test

Using a univariate repeated measures analysis of variance (ANOVA) with random effect, the change in novel object discrimination over time was compared between groups. Differences in novel object discrimination change over the 2 and 7 day time points was considered significant when the *p*-value was less than 0.05. The Shapiro-Wilk test and Levene’s test were used to verify assumptions of normality and homoscedasticity, respectively. Statistical outliers were determined through residual analysis.

#### Western Blot

A one-way ANOVA was used to assess differences in peak areas between treatment groups. Differences were considered significant when the *p*-value was less than 0.05. The Shapiro-Wilk test and Levene’s test were used to verify assumptions of normality and homoscedasticity, respectively. In the event that one of these assumptions was not met, logarithmic or square root transformations were imposed on the data prior to statistical comparisons.

#### HDAC Activity Assay

Samples were compared across treatment groups using a one-way ANOVA comparing activity levels calculated from a standard curve. Differences were considered significant when the *p*-value was less than 0.05. The Shapiro-Wilk test and Levene’s test were used to verify assumptions of normality and homoscedasticity, respectively.

#### IHC

Fluorescent AH3 intensity was normalized to GFAP fluorescent intensity for each image. A one-way ANOVA was used to assess differences in normalized AH3 fluorescence between treatment groups. Differences were considered significant when the *p*-value was less than 0.05. The Shapiro-Wilk test and Levene’s test were used to verify assumptions of normality and homoscedasticity, respectively.

#### RT-PCR

Data was analyzed using Qiagen Data Analysis Web Portal. Fold change was calculated using the previously described 2^ΔΔCt^ method (Livak and Schmittgen, 2001; Schmittgen and Livak, 2008). Fold regulation was calculated as described by manufacturers’ protocol (Qiagen). Positive fold regulation values represent upregulation, while negative values represent downregulation. Due to small sample size (*N* = 2/group), no statistical comparisons were made between groups for the PCR array results.

## Results

### Blast Induced Decline of Recognition Memory

**Figure 2:**
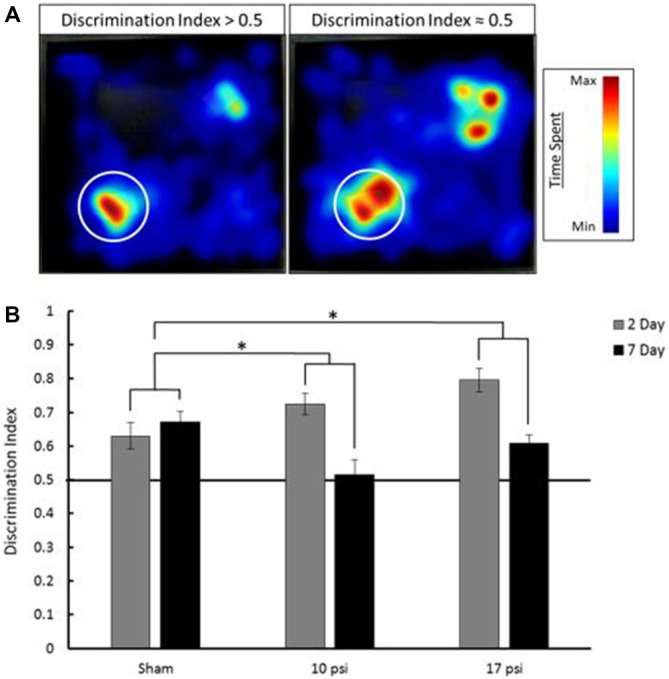
**(A)** Heat map analysis of animal tracking following novel object recognition (NOR) test. Red represents increased time spent and blue represents minimal time spent during trial. Novel object location marked by white circle, familiar object was located in opposite corner. The heat map on the left shows increased time spent at the novel object which shows a discrimination index greater than 0.5 (intact memory). The heat map on the right shows approximately equal time spent at each object which is indicative of a discrimination index approximately equal to 0.5 (memory deficit). **(B)** Average discrimination index for each group at 2 and 7 days following injury. Across time points, each injury group shows a statistically significant decline in memory when compared to sham. *Indicates *p* < 0.05 when compared to sham. Data expressed as mean ± SEM. (*N* = 8/group).

Pathological memory impairments have been well documented following BINT. The NOR test is an established behavior test to evaluate working-memory (Ennaceur and Delacour, 1988). In order to assess working-memory within the first week of injury, the NOR test was administered 2 days and 7 days following injury. Figure 2A shows EthoVision tracking results for the T2 phase of the NOR test. In these images, the colors represent amount of time spent in during the 5 min trial. Blue represents minimal time spent in that area while red represents the maximum amount of time spent during the trial. The left heat map demonstrates increased time spent at the novel object (denoted by the white circle) compared to the familiar object (opposite corner). This behavior is consistent with intact memory and yields a discrimination index greater than 0.5. The image on the right shows approximately equal time spent at both objects. This is indicative of the inability to discern between the novel and familiar object. The image on the right represents memory impairment and the corresponding discrimination index would be approximately equal to 0.5.

The average discrimination index for each group at each time point is summarized in Figure 2B. Sham animals showed intact memory at both the 2 and 7 day time points. Sham animals showed a discrimination index of approximately 0.65. Between time points, these animals showed little change (6.5% increase) in discrimination index. At the 2 day time point both the 10 psi and 17 psi groups also showed intact memory. However, by 7 days following injury, both groups showed severe decline in discrimination index. The 10 psi group showed a 29% decrease in discrimination index. A 23% decrease was observed for the 17 psi group between time points. Decreasing discrimination index is consistent with memory decline. The memory decline between time points was statistically significant when compared to sham for both injury levels (*p* = 0.038, 10 psi; *p* = 0.029, 17 psi). The memory decline was not statistically different between both injury groups (*p* = 0.9463). No reduction in activity levels were observed for any animals during this test.

### Histone Hypo-Acetylation Following Blast Exposure

**Figure 3:**
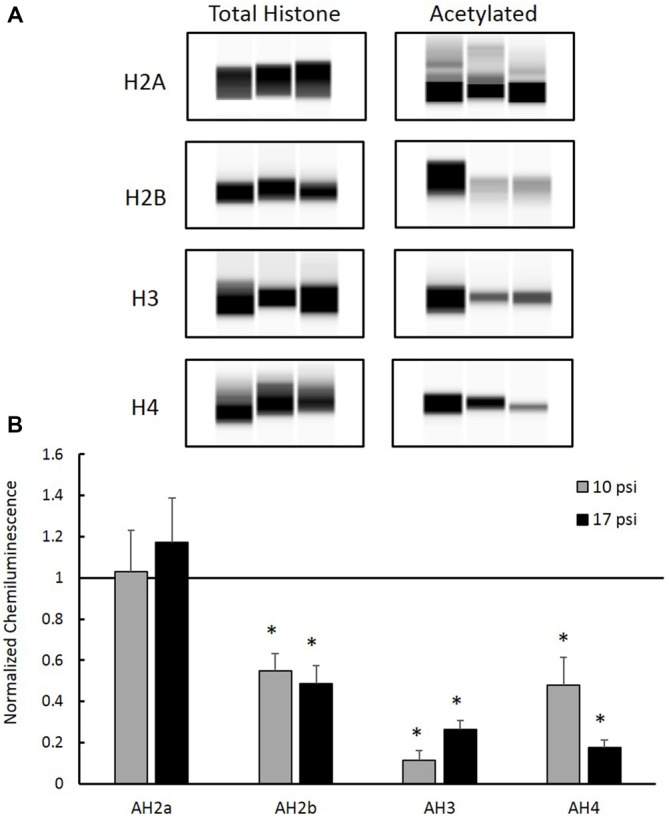
**(A)** Western blot images created by Compass Software (ProteinSimple, Santa Clara, CA, USA) representing modified and total histone expression in the prefrontal cortex (PFC) for each treatment group. No differences in total histone expression was observed for any histone protein. **(B)** Levels of acetyl-H2b (AH2b), acetyl-H3 (AH3), and acetyl-H4 (AH4) were significantly decreased compared to sham. No differences were observed between injury groups. *Indicates *p* < 0.05. Data expressed as mean ± SEM. (*N* = 8/group).

Total histone protein expression levels and acetyl-histone levels were measured using capillary electrophoresis immunoblotting. Representative Western blot images were generated using Compass software (ProteinSimple, Santa Clara, CA, USA) and are shown in Figure 3A. No significant changes were observed between blast groups for any total histone protein expression levels (graph not shown). Figure 3B shows histone acetylation levels for each histone protein normalized to sham. Levels of AH2b, AH3 and AH4 in the PFC decreased following both at 10 psi and 17 psi blast exposure when compared to sham (*p* < 0.05). AH2b showed a 45% decrease following the 10 psi exposure and 51% following the 17 psi exposure. The 10 psi group showed an 89% decrease in AH3 levels while the 17 psi group showed 74% decrease. Levels for AH4 showed 52% and 82% decrease following the 10 and 17 psi exposure, respectively. No changes in acetylation levels were observed between any groups for histone AH2a. No significant changes in acetylation levels were observed between the 10 psi and 17 psi groups for any of the histone proteins.

**Figure 4:**
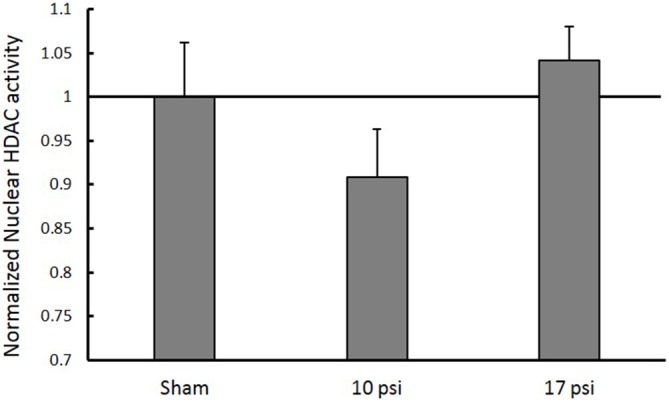
**Normalized nuclear histone deacetylase enzyme (HDAC) activity levels within the PFC.** No significant changes in HDAC activity within the nucleus between any treatment groups. Data expressed as mean ± SEM. (*N* = 8/group).

In order to investigate HDAC enzymes role in histone hypo-acetylation, total HDAC activity was measured from nuclear extracts. The 10 psi group showed slight decreases in HDAC enzyme function and the 17 psi group showed slight increases in HDAC enzyme function. However, there were no statistically significant differences observed between any groups (Figure 4). These results indicate blast exposure did not alter enzyme function at the 7 day time point within the PFC.

### Astrogliosis Involves Decreased Histone Acetylation

**Figure 5:**
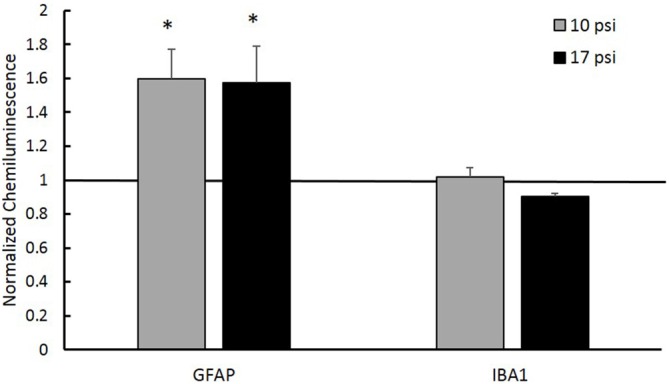
**Western blot analysis showed increased glial fibrillary acidic protein (GFAP) protein levels in the PFC of both injury groups.** A normalized chemiluminescence value of one represents sham expression. This indicates astrogliosis occurring at the 7 day time point. No changes in the microglia marker Ionized Calcium-Binding Adapater Molecule 1 (IBA-1) were observed for either group. *Indicates *p* < 0.05. Data expressed as mean ± SEM. (*N* = 8/group).

In order to test the hypothesis that aberrant histone acetylation may play a role in the chronic inflammatory response following BINT, we investigated levels of inflammatory markers in the PFC. GFAP was used to assess astrocyte activation and IBA-1 was used to investigate microglia response following injury. Simple Western blot analysis showed increased levels of GFAP in the PFC following blast (*p* < 0.05, Figure 5) when compared to sham. Both blast groups showed approximately 60% increases in GFAP protein expression from sham. This suggests increased astrocyte activation. No significant changes were observed in microglia were detected through IBA-1 expression levels.

**Figure 6:**
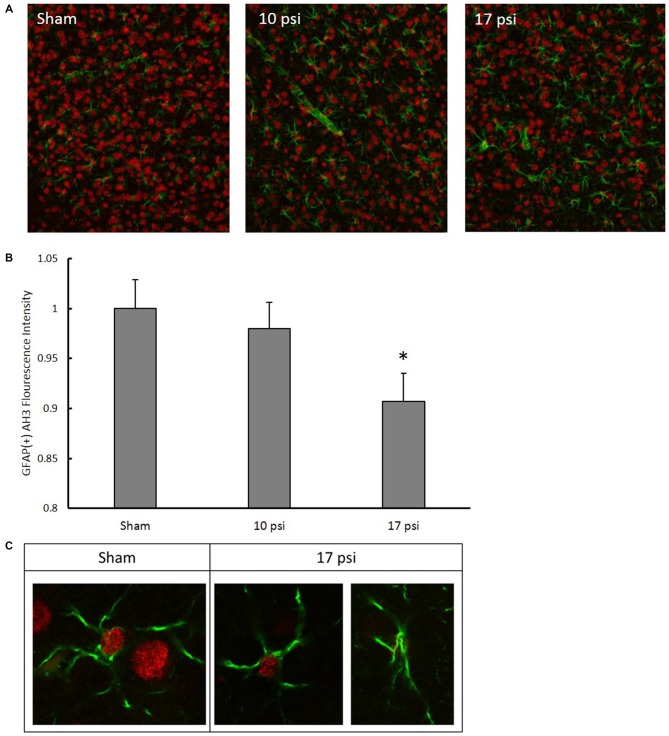
**(A)** Representative images following immunohistochemistry (IHC) analysis. Green fluorescence represents GFAP expression while red represents AH3. In order to isolate, AH3 expression within astrocytes, only co-localized pixels were measured for analysis. **(B)** Decreased expression of AH3 was observed in astrocytes following 17 psi blast. *Indicates *p* < 0.05. Data expressed as mean ± SEM. (*N* = 8/group). **(C)** Images of astrocytes from sham and 17 psi groups taken at 63× to confirm co-localization and demonstrate observed changes in AH3 levels following blast exposure.

After observing significant increases in GFAP levels and since H3 acetylation has previously been implicated in astrocyte activation (Correa et al., 2011; Kanski et al., 2014), we performed a co-localization IHC analysis. For this analysis, we focused specifically in the ACC which has extensive networking with the PFC and is implicated in memory impairment (Lenartowicz and McIntosh, 2005; Einarsson and Nader, 2012). Representative immunofluorescent images are shown in Figure 6A. Red fluorescence represents AH3 while green represents GFAP expression. Diffuse expression of AH3 was observed in each treatment group due to H3 acetylation in several cell types. Only pixels showing GFAP/AH3 co-localization were used for analysis as these pixels represent astrocyte AH3 expression levels. Within these pixels, the average AH3 intensity was measured. AH3 intensity was normalized to GFAP expression in order to account for inflammatory differences between images and groups. Figure 6B summarizes changes observed in GFAP(+) AH3 expression. Blast exposure decreased AH3 expression in astrocytes in both blast groups. However, the change was only significant following the 17 psi exposure (*p* < 0.05). Co-localization was confirmed using confocal microscopy at 63× (Figure 6C). This data suggests that astrocytes exposed to the 10 psi blast undergo histone deacetylation which may play a role in their activation.

### Altered Histone Acetylation and Cytokine Expression

**Figure 7:**
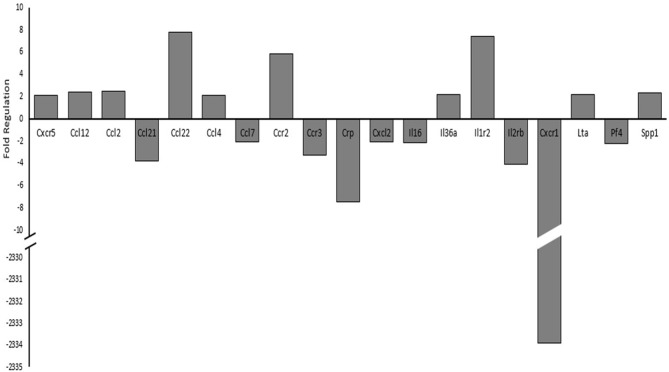
**Changes in cytokine and cytokine receptor mRNA expression levels between sham and 17 psi blast group determined by polymerase chain reaction (PCR) array analysis.** Only genes showing at least a two-fold increase or decrease are shown. Chemokine (C-X-C motif) receptor 5, 1 (CXCR5, CXCR1), Chemokine (C-C motif) ligand 12, 2, 21, 22, 4, 7 (CCL12, CCL2, CCL21, CCL22, CCL4, CCL7), Chemokine (C-C motif) receptor 2, 3 (CCR2, CCR3), C-reactive protein (CRP), Chemokine (C-X-C motif) ligand 2 (CXCL2), Interleukin 16, 36a (IL16, IL36a), Interleukin 1 receptor type 2 (IL1r2), Interleukin 2 receptor beta (IL2rβ), Lymphotoxin Alpha (LTα/TNF-β), Platelet factor 4 (PF4), Secreted phosphoprotein 1 (SPP). (*N* = 2/group).

Since histone acetylation may have a direct impact on transcriptional regulation and is influenced through various intracellular signaling pathways, we investigated cytokine and cytokine receptor mRNA expression levels using PCR arrays (Qiagen, Hilden, Germany). Figure 7 details the genes observed to have a two-fold or greater expression changes between sham and the 17 psi blast group. Ten of the genes included in the PCR array showed increases of two-fold or greater and nine genes showed two-fold or greater decreases in expression. The largest change in expression was observed for Interleukin 8 receptor, alpha (CXCR-1; fold regulation = −2334). Increases in chemokine (C-C motif) ligand 2 (CCL2/MCP1) was observed and have been previously implicated in blast induced neuroinflammation (Cho et al., 2013). The CCL2 receptor, chemokine (C-C motif) receptor 2 (CCR2), also showed a two-fold increase in expression. Interleukin 1 receptor type 2 (IL1r2), which serves as a non-signaling receptor that is important to inhibiting signal from IL-1β, was found upregulating and my result from prolonged stimulation from extracellular IL-1β. Chemokine (C-C motif) ligand 22 (CCL22) expression level was increased 7.7-fold which has observed in other inflammatory disease. Taken together, these results support potential alterations of cytokine signaling that support a pro-inflammatory environment and may involve histone hypoacetylation.

## Discussion

In the work presented here, we observed a time dependent decline in working memory between 2 days and 7 days following blast (Figure 2). The PFC has been shown to play an important role in working memory and memory retrieval. Damage to this region may play a role in the NOR decline (Courtney et al., 1998; Hasegawa et al., 1999; Yoon et al., 2008; Preston and Eichenbaum, 2013). Glial reactivity is important to promote cell survival and neuroprotection but, when uncontrolled, may lead to an environment prone to neurodegeneration and impaired memory (Wyss-Coray and Mucke, 2002, Czerniawski and Guzowski, 2014). Within the PFC, evidence of on-going astrogliosis (Figure 5) was observed 7 days following exposure.

Several studies have proven that histone acetylation is an essential component of memory formation, learning, and synaptic plasticity (Schmitt and Matthies, 1979; Alarcón et al., 2004; Korzus et al., 2004; Levenson et al., 2004; Peleg et al., 2010; Peixoto and Abel, 2013; Lopez-Atalaya and Barco, 2014). Histone hyper-acetylation corresponded with enhanced memory (Levenson et al., 2004; Vecsey et al., 2007), while hypo-acetylation correlated with impaired memory (Valor et al., 2011). These studies have shown that in the absence of injury, memory function and learning was increased in animals showing increased histone acetylation in relevant memory-related brain regions. On the contrary, genetic intervention lead to severe decreases in histone acetylation which manifested as impaired NOR (Valor et al., 2011). Modulation of histone acetylation levels through various therapeutic interventions have shown promise for improving cognitive impairments. For the first time, we report decreased acetylation levels of H2b, H3, and H4 following blast (Figure 3). Using two different blast levels, we observed an average of 65% decrease in histone acetylation for these histones. Analysis of total protein expression levels showed no significant changes among groups, confirming that the observed changes were not a result of altered protein expression but rather acetylation patterns. Since decreased histone acetylation is a critical part of memory function, these changes may provide a molecular basis for the memory decline observed in clinical and pre-clinical studies of brain injury. Moreover, the observed changes to histone acetylation levels likely trigger reorganization of the chromatin. Loss of acetyl groups creates a more positively charged histone tail allowing the DNA wrap around the nucleosome core more tightly (Grant, 2001). This may alter the availability of specific DNA sites to transcription factors triggering altered gene transcription.

In order to provide a better understanding of the impact histone hypoacetylation changes may have in the progression of BINT on the cellular level, we investigated potential interplay with the inflammatory response. Western blot analysis showed elevated GFAP levels in the PFC indicating increased astrocyte activation. Levels of IBA-1 remained unchanged indicating no significant accumulation of microglia within this region. Since increased astrocyte activation was observed and has been previously established as an important part of the injury pathology (Cernak et al., 2011; Kamnaksh et al., 2011; Svetlov et al., 2012; Turner et al., 2013; Sajja et al., 2015, 2016), we sought to investigate histone modifications within astrocytes. Studies have found astrocyte activation correlated with changes to histone acetylation patterns, especially for histone H3 (Correa et al., 2011; Kanski et al., 2014). IHC analysis measured significant decreases in AH3 in astrocytes following injury. Kanski et al. (2014) found that histone acetylation in astrocytes negatively regulates GFAP expression and initiates cytoskeletal reorganization. Here, we describe a similar relationship between histone acetylation and GFAP expression. Since GFAP is a widely accepted marker of astrocyte activation, it is possible that the underlying histone acetylation may be important to the persistent astrocyte activation of BINT.

While histone hypo-acetylation likely contributes, persistent exposure to pro-inflammatory stimuli may cause sustained astrocyte activation. Such stimuli can originate from microglial cytokine secretion. Correa et al. (2011) found that lipopolysaccharide-induced activation of microglia leads to cytokine release that triggers histone hypo-acetylation within astrocytes. We observed no changes in IBA-1 which indicates no significant changes in the PFC microglia population. However, it is possible that the microglia within this region have transitioned to an activated phenotype that leads to secretion of pro-inflammatory cytokines, as has been seen following blast exposure (Kamnaksh et al., 2011; Dalle Lucca et al., 2012; Valiyaveettil et al., 2013; Sajja et al., 2014b). Thus, microglia secretion of pro-inflammatory cytokines may lead to persistent astrocyte activation and histone hypo-acetylation.

Cytokine signaling is an important aspect of the astrocyte response following injury. Several groups have shown cytokine dysregulation following BINT (Kamnaksh et al., 2011; Dalle Lucca et al., 2012; Valiyaveettil et al., 2013; Sajja et al., 2014b). We have demonstrated altered expression levels (greater than two-fold changes) of several other cytokine ligands and receptors (Figure 7). Since histone acetylation directly regulates transcription, the aberrant acetylation patterns have potential to influence the pathological cytokine signaling in astrocytes. The nuclear factor-kappa-light-chain-enhancer of activated B cells (NFκB) signaling pathway is an important pathway involved astrocyte activation and cytokine signaling (Dunn et al., 2002; Emdad et al., 2006; Khorooshi et al., 2008; Thompson and Van Eldik, 2009). NFκB exists in the cytoplasm but translocates into the nucleus upon activation, where it can function as a transcription factor and is involved in the regulation of histone acetylation (Li and Verma, 2002). Activation of NFκB pathways in astrocytes has been shown to lead to the production of CCL2 (Thompson and Van Eldik, 2009). Following PCR array analysis, we observed an approximate 2.5-fold increase of the pro-inflammatory cytokine CCL2 which may indicate NFκB effects (Figure 7). We also observed approximately two-fold increases in IL36a which has been shown to activate NFκB signaling pathways. Therefore, histone acetylation changes following blast may be regulated through NFκB-dependent cytokine signaling and subsequent increased transcription of CCL2 leading to a sustained pro-inflammatory environment. Interestingly, NFκB-dependent histone acetylation is also important for memory function (Lubin, 2011; Federman et al., 2013).

Sajja et al. (2014a) reported diverse neuroprotective efforts involved in the BINT pathology resulting from varied blast magnitudes, similarly our PCR array presents evidence of neuroprotective efforts. Expression of interleukin-8 receptor alpha (CXCR1) showed the largest change (over 2000 fold decrease). CXCR1 expression has been tied to neurons and astrocytes throughout the brain (Danik et al., 2003). Studies have shown that decreases in CXCR1 serve neuroprotective efforts by mitigating the pro-inflammatory effects of IL-8 (Laurén et al., 2010; Sousa et al., 2013). Interestingly, the expression of CXCR1 has also been shown to be regulated through histone acetylation (Baird et al., 2011). Therefore, the observed histone hypo-acetylation following injury may be involved in decreased CXCR1 expression and subsequent neuroprotective efforts by astrocytes.

Decreased histone acetylation underlying the blast-induced inflammatory response presents valuable opportunities for drug intervention. Several studies have focused on administration of HDAC inhibitors following impact-related TBI. These drugs have proven to be efficacious in mitigating the inflammatory response, and restoring memory and functional outcomes. Surprisingly, we did not observe any changes in nuclear HDAC activity levels for either blast group compared to sham (Figure 4). Still, therapeutic intervention of the HDAC/HAT equilibrium can result in regulation of acetylation levels. Despite the absence of pathological HDAC function, inhibiting these enzymes appears to be efficacious in mitigating the pathological cause of histone hypo-acetylation by shifting the HDAC/HAT equilibrium.

Others have investigated the effects of acetate supplementation following lipopolysaccharide-induced neuroinflammation. These studies report the ability to modulate histone acetylation, cytokine regulation and the HDAC/HAT equilibrium (Soliman and Rosenberger, 2011; Soliman et al., 2012). Lastly, increasing histone acetylation has been shown to improve memory, learning and synaptic plasticity (Levenson et al., 2004; Fischer et al., 2007; Fontán-Lozano et al., 2008; Peixoto and Abel, 2013). Taken together, drug intervention of histone acetylation following BINT has promised to mitigate the pathological characteristics including inflammation and memory deficits.

Future studies should seek to address limitations associated with the work presented here. Neuropathology was examined at a single time point; 7 days following injury. Since the pathology of BINT has been shown to involve temporal changes, histone acetylation changes with the progression of the injury from acute to chronic stages would be valuable. The analysis of cytokines presented here was aimed at screening a broad range of cytokines in hopes of elucidating potential downstream histone acetylation effects. The PCR array was carried out with a low sample size. Therefore, further studies should seek to further investigate cytokine dysregulation including CXCR1.

For the first time, we have demonstrated aberrant histone acetylation patterns occurring on histones H2b, H3, and H4 following blast exposure. These changes corresponded with increased astrocyte activation and were further demonstrated within astrocytes through co-localization IHC. Altered expression patterns for several key chronic inflammatory cytokine/cytokine receptors were observed. Altered cytokine expression may influence NFκB pathways and play a role in pathological gene expression through improper regulation of histone acetylation. Finally, histone hypoacetylation in the PFC was associated with evidence of memory decline at the 7 day time point following blast. Future studies need to focus on the mechanisms leading to the observed histone deacetylation and the role hypo-acetylation plays in sustained astrocyte reactivity and transcription to fully establish the cellular response to blast. A better understanding of histone acetylation and BINT would be valuable for future therapeutic design.

## Author Contributions

PJV designed and conceived the blast model. PJV and ZSB performed all blast tests. ZSB conducted behavioral testing. ZSB and MBG conducted *ex vivo* analysis.

## Funding

The project was supported by the Department of Veterans Affairs RRD award #RX001104-01.

## Conflict of Interest Statement

The authors declare that the research was conducted in the absence of any commercial or financial relationships that could be construed as a potential conflict of interest.

## Abbreviations

ABS: advanced blast simulator
ACC: anterior cingulate cortex
AH2a: acetyl-H2a
AH2b: acetyl-H2b
AH3: acetyl-H3
AH4: acetyl-H4
ANOVA: analysis of variance
BBB: blood brain barrier
BCA: Bradford colorimetric assay
BINT: blast induced neurotrauma
CCL2: chemokine (C-C motif) ligand 2
CCL22: chemokine (C-C motif) ligand 22
CCR2: chemokine (C-C motif) receptor 2
CXCR1: interleukin 1 receptor, alpha
DAPI: 6-diamidino-2-pheylindole
HAT: histone acetyltransferase enzyme
HDAC: histone deacetylase enzyme
IBA-1: ionized calcium-binding adapter molecule 1
IFNγ: interferon gamma
IHC: immunohistochemistry
IL1β: interleukin 1, beta
IL1r2: interleukin 1 receptor, type 2
IL8: interleukin 8
NFκB: nuclear factor-kappa-light-chain-enhancer of activated B cells
NOR: novel object recognition
PBS: phosphate buffered saline
PFC: prefrontal cortex
RT-PCR: reverse transcription polymerase chain reaction
TBI: traumatic brain injury
TNFα: tumor necrosis factor, alpha.
